# Comparison of hamstring transfer with hamstring lengthening in ambulatory children with cerebral palsy: further follow-up

**DOI:** 10.1007/s11832-014-0626-8

**Published:** 2014-11-28

**Authors:** Camila De Mattos, K. Patrick Do, Rosemary Pierce, Jing Feng, Michael Aiona, Michael Sussman

**Affiliations:** 1 Hospital Estadual da Criança, Rio de Janeiro, Brazil; 2 Shriners Hospitals for Children—Portland, 3101 SW Sam Jackson Park Road, Portland, OR 97239 USA

**Keywords:** Cerebral palsy, Hamstring lengthening, Hamstring transfers, Motion analysis, Long-term study

## Abstract

**Background:**

Overactivity or contractures of the hamstring muscles in ambulatory children with cerebral palsy (CP) can lead to either a jump gait (knee flexion associated with ankle plantar flexion) or a crouch gait (knee flexion associated with ankle dorsiflexion). Hamstring lengthening is performed to decrease stance knee flexion. However, this procedure carries the potential risk of weakening hip extension power as well as recurrence over time; therefore, surgeons have adopted a modified procedure wherein the semitendinosus and gracilis are transferred above the knee joint, along with lengthening of the semimembranosus and biceps femoris.

**Purpose:**

The purpose of our study is to evaluate the differences between hamstring lengthening alone (HSL group) and hamstring lengthening plus transfer (HST group) in the treatment of flexed knee gait in ambulatory children with CP. We hypothesized that recurrence of increased knee flexion in the stance phase will be less in the HST group at long-term follow-up, and hip extensor power will be better preserved.

**Methods:**

Fifty children with CP who underwent hamstring surgery for flexed knee gait were retrospectively reviewed. All subjects underwent a pre-operative gait study, a follow-up post-operative gait study, and a long-term gait study. The subjects were divided into two groups; HSL group (18 subjects) or HST group (32 subjects). The mean age at surgery was 9.9 ± 3.3 years. The mean follow-up time was 4.4 ± 0.9 (2.7–6.3) years.

**Results:**

On physical examination, both groups showed improvement in straight leg raise, knee extension, popliteal angle, and maximum knee extension in stance at the first post-op study, and maintained this improvement at the long-term follow-up, with the exception of straight leg raise, which slightly worsened in both groups at the final follow-up. Both groups improved maximum knee extension in stance at the initial follow-up, and maintained this at the long-term follow-up. Only the HST group showed significant (*p* < 0.05) improvement in the peak hip extension power in stance at the first post-op study, and this increased further at the final follow-up. In the HSL group, there was an initial slight decrease in the hip extension power, which subsequently increased to pre-operative values at the long-term study. Only the HST group showed increase of the average anterior pelvic tilt at the long-term follow-up study, although this was small in magnitude. There were two subjects who developed knee recurvatum at the post-op study, and both were in the HST group.

**Conclusions:**

There is no clear benefit in regards to recurrence when comparing HST to HSL in the long term. In both HSL and HST, there was reduction of stance phase knee flexion in the long term, with no clear advantage in either group. Longer follow-up is needed for additional recurrence information. There was greater improvement of hip extension power in the HST group, which may justify the additional operative time of the transfer.

**Significance:**

This study helps pediatric orthopedic surgeons choose between two different techniques to treat flexed knee gait in patients with CP by showing the long-term outcome of both procedures.

## Introduction

Hamstring tightness or overactivity in ambulatory children with cerebral palsy (CP) can lead to either a jump gait (knee flexion associated with ankle plantar flexion) or a crouch gait (knee flexion associated with ankle dorsiflexion) [1]. Hamstring lengthening is performed to increase knee extension during the stance phase.

In 1952, Eggers advocated the transfer of all hamstrings to the femoral condyles [2], but this was later seen to cause recurvatum in a significant number of patients, leaving surgeons to avoid transferring hamstring tendons for many decades. Sutherland et al. [3] as well as Ray and Ehrlich [4] reported transfer of the gracilis and semitendinosus tendons laterally in hopes of improving internal rotation as well as increase knee flexion.

Sung et al. [5] as well as Selber et al. [6] have more recently reintroduced transfer of the semitendinosus and gracilis to the adductor magnus tendon. Selber et al. found that the transfers limited step length, particularly in the higher functioning patients, while Sung et al. reported no negative effects from the transfers. However, both groups felt that the transfer was valuable.

In an earlier study, we showed that transferring the gracilis and semitendinosus to the adductor tubercle had similar benefits to lengthening alone on knee function when compared to traditional lengthening, but, in addition, preserved hip extension power [4]. This study examines the longer term data of outcomes of patients undergoing hamstring lengthening plus transfers (HST) compared with a cohort who underwent traditional hamstring lengthening alone surgery (HSL).

During traditional hamstring procedures, the semitendinosus and gracilis are tenotomized or lengthened instead of transferred. This may possibly allow these muscles to reattach and shorten, which increases the likelihood for recurrence. Therefore, we hypothesized that recurrence of increased knee flexion in the stance phase would be less in the HST group than in the HSL group.

## Materials and methods

A retrospective review was conducted on all patients who had gait studies performed from July 1997 to June 2013 prior to having either hamstring lengthening surgery or hamstring lengthening surgery in combination with transfer of the distal hamstring tendons. Many of the patients in this study took part in an earlier study but were not included in this study due to our inability to obtain longer term follow-up on all the patients from the first study [7]. Institutional Review Board approval was obtained. In general, HSL was performed prior to 2004, while HST was introduced after this time and became the standard procedure based on the perceived theoretical benefits of this modification.

**Fig. 1:**
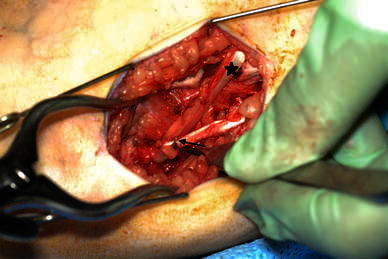
The semitendinosus (*star*) and gracilis (*arrow*) tendons transferred to the adductor tendon distally

Patients were excluded from this study if they had concomitant or subsequent femoral extension osteotomy, history of selective dorsal rhizotomy, or significant extrapyramidal involvement. Children who had concomitant or subsequent iliopsoas lengthening were included in the study, but their data were excluded from the hip power analysis because this procedure may directly influence hip power. The HSL group underwent traditional lengthening, in which the gracilis and semitendinosus tendon were divided or underwent intramuscular lengthening, in which the tendon fibers were divided proximal to the musculotendinous junction, so the tendon ends were separated but continuity was maintained by the surrounding muscle, or, in some cases, the semitendinosus was lengthened in a Z-fashion. An aponeurotic intramuscular lengthening of the semimembranosus was performed in all cases. Lateral hamstring lengthening was added at the surgeon’s discretion, in which case an aponeurotic intramuscular lengthening of the biceps femoris was performed. In the HST group, the semitendinosus and gracilis tendons were transferred to the adductor magnus tendon proximal to its insertion on the adductor tubercle (Fig. 1) with aponeurotic intramuscular lengthening of the semimembranosus. The transferred tendons were attached firmly but not tightly to the attachment site to avoid restriction of hip flexion. Biceps femoris aponeurotic intramuscular lengthening was added for some patients per the surgeon’s choice [7].

Post-operatively, patients were kept in knee immobilizers for comfort for 1–3 days with hip flexion to protect against sciatic nerve stretch. Patients were mobilized beginning on the first post-op day and were allowed out of the knee immobilizers as comfort allowed. By the second or third post-op day, knee immobilizers were used at night only. Most patients wore rigid 90° ankle–foot orthoses for weight-bearing activities, and some patients wore ground reaction ankle–foot orthoses [7].

All patients underwent a pre-operative gait study, a follow-up post-operative gait study at approximately 1 year post-op, and a long-term gait study (2.7–6.3 years). Long-term studies were either retrospective as part of ongoing care (42 subjects) or collected prospectively from volunteer subjects meeting the qualifying timeline proposed of 3.5–6.5 years post-op (eight subjects).

A total of 50 children (38 males and 12 females) ranging in age from 5 to 17 years old (9.9 ± 3.3 years at the time of surgery) were included in this study. There were 32 patients in the HST group and 18 patients in the HSL group. The average long-term follow-up time from pre-op was 4.4 ± 0.9 years for both groups. A detailed description of the patients is shown in Table 1.

**Table 1:** Subject characteristics

Characteristic	HSL (*n* = 18)	HST (*n* = 32)
Diagnosis		
Monoplegia	0	1
Spastic diplegia	11	24
Spastic triplegia	2	4
Spastic quadriplegia	2	2
Hemiplegia	2	2
Gender		
Male	17	21
Female	1	11
Age at surgery (years)	11.6 ± 3.4	10.5 ± 2.9
First follow-up time (years)	1.1 ± 0.3	1.2 ± 0.3
Long-term follow-up time (years)	4.3 ± 0.9	4.5 ± 0.89
GMFCS level		
I	8	8
II	4	14
III	6	10

Data collection included: age, gender, Gross Motor Function Classification System (GMFCS) level, age at surgery, physical examination measurements, and kinematic and kinetic data from 3D computerized gait analysis. Operative notes from the hamstring surgeries and concomitant surgeries were collected. All surgeries were performed by one of two experienced pediatric orthopedic surgeons at the same institution.

Vicon (Vicon Motion Systems Ltd., UK) software was used to calculate the kinematic and kinetic data. A two-tailed paired *t*-test with the significance level set at 0.05 was used to detect differences between pre-op and post-op measurements. All statistical analysis of the data was done only for the right legs to meet the statistical requirement for independence, unless the hamstring surgery was only performed on the left leg.

## Results

**Fig. 2:**
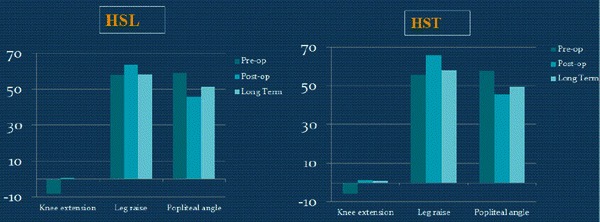
Pre-op, post-op, and long-term static measurements of the two groups

There were 16 patients at GMFCS level I, 18 patients at level II, and 16 patients at level III. Most patients had multilevel releases, and some may have had prior adductor and/or iliopsoas lengthening. Surgeries performed concomitantly with hamstring surgeries are shown in Table 2. The static knee extension and popliteal angle were both improved at the first post-op study and maintained at final follow-up, while straight leg raise was improved at the first post-op study but returned close to pre-op values by the final follow-up (Tables 3 and 4; Fig. 2).

**Table 2:** Concomitant surgeries

Type of concomitant procedure	Number of single procedures
Rectus transfer	30
Adductor lengthening	18
Gastrocnemius lengthening (Strayer procedure)	16
Psoas lengthening	15
Removal of hardware	11
Achilles tendon lengthening	5
Derotational osteotomy of the femur	4
Posterior tibialis lengthening	3
Subtalar fusion	3
Shortening osteotomy of the femur	1
Derotational osteotomy of the tibia	1
Plantar fascia release	1
Total number of concomitant procedures	108
Total number of patients with concomitant surgeries	41

**Table 3:** Pre- and post-operative measurements for the HSL and HST groups

	HSL				HST			
	Pre	Post	Change	*p* value	Pre	Post	Change	*p* value
Physical examination	*n* = 18				*n* = 32			
Straight leg raise (°)	57.8 ± 7.7	63.3 ± 8.2	5.6 ± 10.8	<0.05	55.8 ± 9.3	65.9 ± 6.8	10.2 ± 11.4	<0.05
Knee extension (°)	−8.3 ± 8.6	0.3 ± 5.6	8.6 ± 7.8	<0.05	−5.6 ± 8.5	1.3 ± 5.6	6.9 ± 8.7	<0.05
Popliteal angle (°)	58.9 ± 14.4	45.6 ± 9.7	−13.3 ± 14.5	<0.05	57.7 ± 10.7	45.6 ± 9.7	−14.9 ± 13.9	<0.05
Kinematics	*n* = 18				*n* = 32			
Average pelvic tilt (°)	16.7 ± 7.1	21.6 ± 7.6	4.9 ± 9.2	<0.05	14.1 ± 7.0	20.1 ± 5.8	5.9 ± 8.2	<0.05
Minimum knee flexion in stance (°)	22.4 ± 13.3	9.5 ± 10.4	−13.0 ± 10.8	<0.05	24.9 ± 11.9	7.2 ± 10.4	−17.7 ± 13.2	<0.05
Minimum hip flexion in stance (°)	10.1 ± 11.0	7.5 ± 8.8	−2.6 ± 8.6	0.21	6.9 ± 9.4	4.3 ± 10.2	−2.6 ± 9.1	0.11
Kinetics	*n* = 8				*n* = 16			
Peak hip power in stance (W/Kg)	1.0 ± 0.4	0.9 ± 0.5	−0.1 ± 0.7	0.66	1.0 ± 0.5	1.2 ± 0.5	0.2 ± 0.3	<0.05

**Table 4:** Pre-operative and long-term measurements for the HSL and HST groups

	HSL				HST			
	Pre	Long-term	Change	*p* value	Pre	Long-term	Change	*p* value
Physical examination	*n* = 18				*n* = 32			
Straight leg raise (°)	57.8 ± 7.7	58.3 ± 9.4	0.3 ± 10.1	0.90	55.8 ± 9.3	57.9 ± 6.2	2.2 ± 11.8	0.30
Knee extension (°)	−8.3 ± 8.6	0.0 ± 8.4	8.3 ± 10.7	<0.05	−5.6 ± 8.5	0.8 ± 8.6	6.3 ± 10.9	<0.05
Popliteal angle (°)	58.9 ± 14.4	51.1 ± 12.3	−7.8 ± 14.9	<0.05	57.7 ± 10.7	49.4 ± 10.1	−8.3 ± 15.1	<0.05
Kinematics	*n* = 18				*n* = 32			
Average pelvic tilt (°)	16.7 ± 7.1	19.6 ± 7.3	2.6 ± 10.3	0.30	14.1 ± 7.0	19.3 ± 7.9	5.4 ± 8.6	<0.05
Minimum knee flexion in stance (°)	22.4 ± 13.3	10.8 ± 12.1	−11.7 ± 12.5	<0.05	24.9 ± 11.9	12.5 ± 14.3	−12.4 ± 17.6	<0.05
Minimum hip flexion in stance (°)	10.1 ± 11.0	8.4 ± 7.9	−1.7 ± 9.7	0.45	6.9 ± 9.4	8.3 ± 12.2	1.5 ± 11.6	0.49
Kinetics	*n* = 8				*n* = 16			
Peak hip power in stance (W/Kg)	1.0 ± 0.4	1.2 ± 0.4	0.2 ± 0.6	0.51	1.0 ± 0.5	1.4 ± 0.7	0.4 ± 0.7	<0.05

**Fig. 3:**
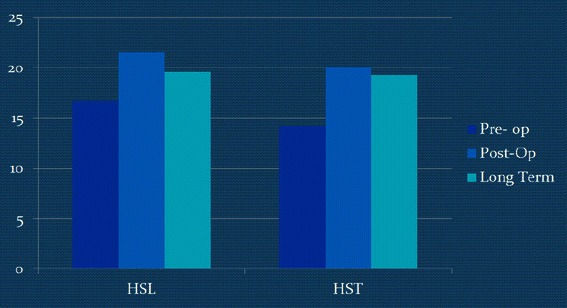
Pre-op, post-op, and long-term pelvic tilt values of the two groups

Pelvic tilt increased in both groups, but only the HST group had a significant difference from pre-op levels at the final follow-up, although the magnitude of the difference was only 2.8° (Tables 3 and 4; Fig. 3).

**Fig. 4:**
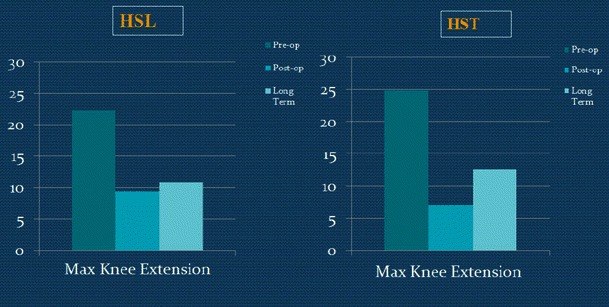
Pre-op, post-op, and long-term dynamic maximum knee extension of the two groups

Dynamic maximum knee extension in stance was decreased in both groups at the first post-op study, and this was maintained at the final follow-up (Tables 3 and 4; Fig. 4).

**Fig. 5:**
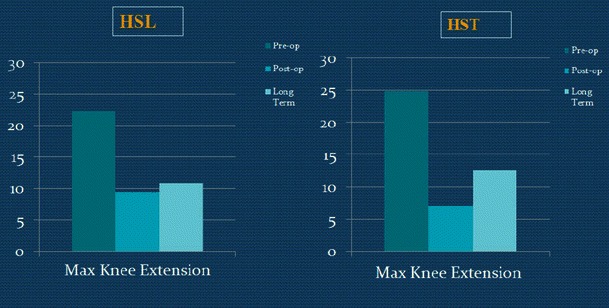
Pre-op, post-op, and long-term dynamic maximum hip extension of the two groups

Maximum hip extension in stance for both groups did not change after surgery (Tables 3 and 4; Fig. 5).

**Fig. 6:**
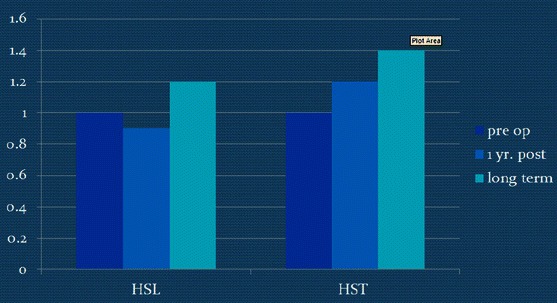
Pre-op, post-op, and long-term dynamic maximum hip power of the two groups

Peak hip extension power improved at the first post-op study in the HST group, while there was mild loss of power in the HSL group (Table 3; Fig. 6). Both groups showed improvement between 1 year post-op and the final follow-up, with the greatest overall improvement in the HST group (Table 4; Fig. 6). Two patients in the transfer group developed recurvatum.

Eleven subjects (four in the HSL group and seven in the HST group) had only medial hamstring lengthenings. However, the results were similar even after these subjects were removed from the statistical analysis.

## Discussion

In typically developing children, the hamstring muscles, with the exception of the short head of the biceps, are active in an eccentric mode in the late swing phase in order to decelerate the forward flexing limb. They continue to be active concentrically in early stance as the hip begins to extend, during which time they act as accessory hip extensors serving to move the center of mass forward over the stance phase limb [8] (providing about 30 % of the hip extension power) [9]. In many CP patients, the spastic hamstrings are active throughout the entire stance phase, leading to persistent knee flexion as well as shortened stride length, since they restrict maximum hip flexion [10]. The hamstrings also become shortened over time, leading to knee flexion contracture. This problem usually does not begin to manifest before the age of 5 years and, once it appears, it generally worsens with time.

Surgical lengthening of the hamstrings to address this problem is commonly performed by pediatric orthopedic surgeons and it is a validated procedure [11–13].

Transfer of the semitendinosus and gracilis to the adductor tendon insertion area was introduced in hope of preserving what was thought to be loss of hip extension power, as well as reduce recurrence of hamstring tightness. The current study showed improvement in knee extension in stance, but no difference between groups was seen at either post-op interval (Tables 3 and 4) by 3D kinematic analysis. However, hip extension power was improved in the HST group at the 1-year post-op assessment, while there was a decrease in the HSL group, but both groups showed greater power between the first post-op and the final follow-up gait analyses (Tables 3 and 4). Many surgeons are concerned that hamstring lengthening may cause loss of hip extension power, since they contribute 25–30 % of the overall hip extension power [9]. However, we found that the assumption that hip extension power is lost in the long term following hamstring lengthening is invalid. Thus, the “surgical dose” that we used was sufficient to yield improvements in objective gait parameters without having any adverse effects.

Selber et al. [6] suggested transferring the semitendinosus to the adductor tubercle, but they warned that it could restrict hip flexion. In our study, we transferred both the semitendinosus as well as the gracilis to the adductor tendon and found no significant difference in the minimum hip flexion in the stance phase for both the HST and HSL groups in the short- and long-term follow-up. However, we avoided attaching the transfers tightly and attached them with only a small amount of tension, which avoided restriction of hip flexion.

Biceps femoris lengthening was added at the surgeon’s discretion to improve maximum knee extension during the stance phase, usually in patients with severe crouch and knee flexion contractures [12]. There is a concern that biceps lengthening may cause excessive weakening, but we felt that leaving it intact would compromise the result and lead to insufficient correction. We don’t have the final answer on this, but there does not seem to be excessive weakening in those patients who had biceps lengthening.

**Fig. 7:**
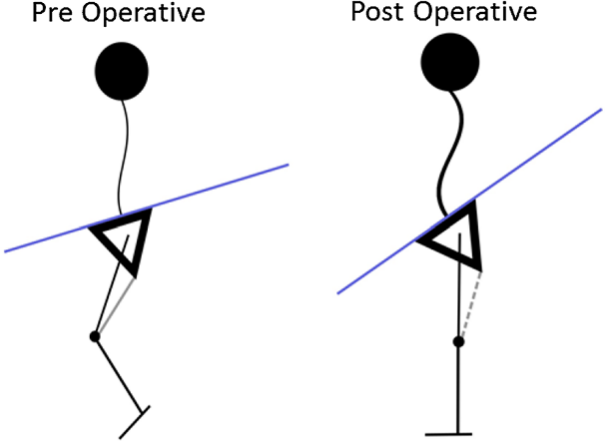
Diagrammatic representation of a stick figure of a patient with tight hamstrings before and after HSL or HST: **a** pre-operative, **b** post-operative. The relationship of the femur to the pelvis is unchanged, but by making the femur more vertical, the pelvic tilt must increase

Hamstring lengthening, with or without transfer, does cause a minimal increase in anterior pelvic tilt and, thus, an increase in lumbar lordosis [14]. Pelvic tilt is influenced by muscle function in the lumbar spine and pelvic girdle musculature, as well as the hamstrings. We believe that an increase in pelvic tilt is inevitable when flexed knee gait is corrected, since the oblique femur during stance becomes more vertical, thus rotating the pelvis in more of an anterior tilt, unless hip extension is reciprocally increased [4]. Therefore, if the pelvic tilt remains the same or increases mildly, it means that there has actually been an improvement on the pelvic position (Fig. 7) [4]. Both groups showed a small increase in average pelvic tilt in the first post-op study, but only in the HST group was there a small but significant increase in the long term. Longer follow-up is necessary to see if this trend continues with time. Metaxiotis et al. [15] reported improvements in dynamic knee function without an increase in anterior pelvic tilt after transferring the semitendinosus to the medial origin of the gastrocnemius.

Recurvatum has been shown to be a complication of hamstring surgery [3]. In our study, we had two patients in the HST group and none in the HSL group that showed recurvatum. However, due to the small number of subjects, no conclusion should be drawn from these data.

Ma et al. [16] demonstrated good static and dynamic outcomes after hamstring transfer surgery with no recurvatum in any patient, but there was no cohort for comparison with the traditional lengthening.

This study confirms that HST is a safe procedure, but we did not find a clear benefit in regards to recurrence between HST and HSL during the follow-up period of 2.7–6.3 years. In both procedures, there was improved stance phase knee flexion in the longer term follow-up compared to pre-op values. The one major difference between the groups is a greater improvement of hip extension power in the HST group. A longer term follow-up is needed in order to obtain additional information about the recurrence of flexed knee gait.

This study helps pediatric orthopedic surgeons choose between two different techniques to treat flexed knee gait in patients with CP by showing a longer follow-up assessment of gait for both procedures.

## References

[1] Winters TF, Gage JR, Hicks R (1987). Gait patterns in spastic hemiplegia in children and young adults. J Bone Joint Surg Am.

[2] Eggers GWN (1952). Transplantation of hamstring tendons to femoral condyles in order to improve hip extension and to decrease knee flexion in cerebral spastic paralysis. J Bone Joint Surg Am.

[3] Sutherland DH, Schottstaedt ER, Larsen LJ, Ashley RK, Callander JN, James PM (1969). Clinical and electromyographic study of seven spastic children with internal rotation gait. J Bone Joint Surg Am.

[4] Ray RL, Ehrlich MG (1979). Lateral hamstring transfer and gait improvement in the cerebral palsy patient. J Bone Joint Surg Am.

[5] Sung KH, Chung CY, Lee KM, Akhmedov B, Lee SY, Choi IH, Cho TJ, Yoo WJ, Park MS (2013). Long term outcome of single event multilevel surgery in spastic diplegia with flexed knee gait. Gait Posture.

[6] Selber P, Kerr Graham H, Gage J (2012). Re: Feng L, Do P, Aiona M, Feng J, Pierce R, Sussman M (2012) Comparison of hamstring lengthening with hamstring lengthening plus transfer for the treatment of flexed knee gait in ambulatory patients with cerebral palsy. J Child Orthop 6:229–235. J Child Orthop.

[7] Feng L, Patrick Do K, Aiona M, Feng J, Pierce R, Sussman M (2012). Comparison of hamstring lengthening with hamstring lengthening plus transfer for the treatment of flexed knee gait in ambulatory patients with cerebral palsy. J Child Orthop.

[8] Perry J, Newsam C, Sussman MD (1992). Function of the hamstrings in cerebral palsy. The diplegic child.

[9] Waters RL, Perry J, McDaniels JM, House K (1974). The relative strength of the hamstrings during hip extension. J Bone Joint Surg Am.

[10] Aiona MD, Sussman MD (2004). Treatment of spastic diplegia in patients with cerebral palsy: Part II. J Pediatr Orthop B.

[11] Baumann JU, Ruetsch H, Schürmann K (1980). Distal hamstring lengthening in cerebral palsy. An evaluation by gait analysis. Int Orthop.

[12] Kay RM, Rethlefsen SA, Skaggs D, Leet A (2002). Outcome of medial versus combined medial and lateral hamstring lengthening surgery in cerebral palsy. J Pediatr Orthop.

[13] Rodda JM, Graham HK, Nattrass GR, Galea MP, Baker R, Wolfe R (2006). Correction of severe crouch gait in patients with spastic diplegia with use of multilevel orthopaedic surgery. J Bone Joint Surg Am.

[14] DeLuca PA, Ounpuu S, Davis RB, Walsh JH (1998). Effect of hamstring and psoas lengthening on pelvic tilt in patients with spastic diplegic cerebral palsy. J Pediatr Orthop.

[15] Metaxiotis D, Wolf S, Doederlein L (2004). Conversion of biarticular to monoarticular muscles as a component of multilevel surgery in spastic diplegia. J Bone Joint Surg Br.

[16] Ma FYP, Selber P, Nattrass GR, Harvey AR, Wolfe R, Graham HK (2006). Lengthening and transfer of hamstrings for a flexion deformity of the knee in children with bilateral cerebral palsy: technique and preliminary results. J Bone Joint Surg Br.

